# Modeling the heterogeneity in risk of progression to Alzheimer's disease across cognitive profiles in mild cognitive impairment

**DOI:** 10.1186/alzrt168

**Published:** 2013-03-06

**Authors:** Curtis Tatsuoka, Huiyun Tseng, Judith Jaeger, Ferenc Varadi, Mark A Smith, Tomoko Yamada, Kathleen A Smyth, Alan J Lerner, The Alzheimer's Disease Neuroimaging Initiative

**Affiliations:** 1 Department of Neurology, Case Western Reserve University, 11100 Euclid Avenue, Cleveland, OH 44106 USA; 2 Neurological Institute, University Hospitals Case Medical Center, 3619 Park East Drive, Beachwood, OH 44122 USA; 3 Department of Epidemiology and Biostatistics, Case Western Reserve University, 10900 Euclid Avenue, Cleveland, OH 44106 USA; 4 Department of Human Development, Teachers College, Columbia University, 525 West 120th Street, New York, NY 10027 USA; 5 AstraZeneca Pharmaceuticals, Clinical Development, Neuroscience, 1800 Concord Pike, Wilmington, DE 19807 USA; 6 Department of Psychiatry and Behavioral Sciences, Albert Einstein College of Medicine, 1300 Morris Park Avenue, Bronx, NY 10461 USA; 7 Tanar Software, Hunting Valley, OH 44022 USA; 8 Department of Pathology, Case Western Reserve University, 2103 Cornell Road, Cleveland, OH 44106 USA

## Abstract

**Introduction:**

Heterogeneity in risk of conversion to Alzheimer's disease (AD) among individuals with mild cognitive impairment (MCI) is well known. Novel statistical methods that are based on partially ordered set (poset) models can be used to create models that provide detailed and accurate information about performance with specific cognitive functions. This approach allows for the study of direct links between specific cognitive functions and risk of conversion to AD from MCI. It also allows for further delineation of multi-domain amnestic MCI, in relation to specific non-amnestic cognitive deficits, and the modeling of a range of episodic memory functioning levels.

**Methods:**

From the Alzheimer's Disease Neuroimaging Initiative (ADNI) study, conversion at 24 months of 268 MCI subjects was analyzed. It was found that 101 of those subjects (37.7%) converted to AD within that time frame. Poset models were then used to classify cognitive performance for MCI subjects. Respective observed conversion rates to AD were calculated for various cognitive subgroups, and by APOE e4 allele status. These rates were then compared across subgroups.

**Results:**

The observed conversion rate for MCI subjects with a relatively lower functioning with a high level of episodic memory at baseline was 61.2%. In MCI subjects who additionally also had relatively lower perceptual motor speed functioning and at least one APOE e4 allele, the conversion rate was 84.2%. In contrast, the observed conversion rate was 9.8% for MCI subjects with a relatively higher episodic memory functioning level and no APOE e4 allele. Relatively lower functioning with cognitive flexibility and perceptual motor speed by itself also appears to be associated with higher conversion rates.

**Conclusions:**

Among MCI subjects, specific baseline cognitive profiles that were derived through poset modeling methods, are clearly associated with differential rates of conversion to AD. More precise delineation of MCI by such cognitive functioning profiles, including notions such as multidomain amnestic MCI, can help in gaining further insight into how heterogeneity arises in outcomes. Poset-based modeling methods may be useful for providing more precise classification of cognitive subgroups among MCI for imaging and genetics studies, and for developing more efficient and focused cognitive test batteries.

## Introduction

There is increasing interest in the recognition and treatment of prodromal stages of Alzheimer's disease (AD), especially mild cognitive impairment (MCI). MCI is viewed as a state between normal cognitive functioning and dementia. Those with MCI are characterized as exhibiting mild problems with memory and/or other cognitive functions, while still being able to perform daily life activities normally or nearly so [1]. Despite having a higher overall risk of developing AD [1], conversion outcomes among those with MCI are quite heterogeneous. Only about 15% of these individuals convert to AD per year; many never convert, and some revert to normal cognition [2]. This heterogeneity makes it difficult to design efficient trials of agents designed to delay or prevent progression from MCI to AD or to interpret the outcomes of these trials [3], or to evaluate potential AD biomarkers. Hence, there is a clear need to better delineate cognitive phenotypes in MCI.

Although MCI subgroups that reflect deficit heterogeneity, such as amnestic single domain MCI, amnestic multidomain MCI, and non-amnestic multidomain MCI [4] have been developed, they lack specificity in the particular cognitive functions that are impaired in each subgroup. This type of specification is challenging because neuropsychological (NP) response data are complex. It can be difficult to isolate a deficit in a particular cognitive function, since performing well on most NP measures requires tapping into several cognitive functions, and it is often not possible to design tests that tap one cognitive domain to the exclusion of all others. For example, it is possible to perform poorly on a verbal list-learning task as a result of impaired attention or word fluency, and in the absence of an amnestic disturbance. Hence, if an individual performs poorly on a given measure, it may be difficult to pinpoint exactly which function is impaired.

Subscales are commonly used in an attempt to improve specificity in analysis of NP assessment data. For instance, subscales can be derived from factor analysis through the use of factor scores. However, scale-based approaches are generally limited by the assumption of a direct correspondence between a subscale score and an associated function. Poor performance on a subscale is interpreted as indicating a deficit in the function the subscale is purported to measure, even if the poor performance is due to deficits in functions not associated with the subscale. This makes it difficult to link NP test performance to specific functional deficits, and hence to identify cognitive phenotypes that can be linked to outcomes such as conversion from MCI to AD.

Use of total scores on multi-item measures, such as the Alzheimer's disease assessment scale-cognitive (ADAS-Cog) measure [5] or the mini-mental status exam (MMSE) [6], as a basis for cognitive phenotyping is also problematic. Total scores represent a (weighted) sum of response scores from items assessing several cognitive domains. However, the same total score can be derived from a range of different response patterns, without regard to the cognitive functions being assessed by each item. In this sense, items are viewed as interchangeable, even though the cognitive targets of assessment for the items can vary considerably. Because a wide range of response patterns and cognitive interpretations can give rise to the same score, resulting phenotypes lack specificity.

### The partially ordered set (poset) modeling approach to interpreting NP data

Poset models serve as a basis for novel methods tailored for classifying the performance (that is, functioning) levels of subjects with respect to specific cognitive functions. Classification is conducted based on observed responses to NP measures. Each measure is associated with specific cognitive functions that are involved in performing well on that measure, so that the ties between observed responses and functioning levels are clear. This approach is feasible because posets have essential statistical convergence properties such as assuring that a subject's state is identified accurately with sufficient measurement, even in the presence of measures that are associated with multiple functions. Theoretically derived validation tools are available as well. Statistical theory and data-analytic frameworks for the poset approach have been established in Tatsuoka and Ferguson (2003) [7] and Tatsuoka (2002) [8].

In this paper, our goal is to demonstrate that posets can improve our understanding of MCI heterogeneity. The modeling results in the development of states associated with profiles of cognitive functioning that summarize performance levels for each of the cognitive functions being tested by a given NP battery. Hence, a state can be viewed as similar to a diagnostic classification in which the diagnosis represents a particular pattern of cognitive strengths and weaknesses. States are ordered by comparing the associated performance levels for each of the functions included in the analysis. One state is considered greater than a second state if its associated performance level on at least one function is strictly higher than the performance level for the second state, and its performance levels for all other functions are at least as high. However, posets are flexible in that it is not necessary that one state be greater than another, in other words, the states can be partially ordered. This arises when one state in comparison to another state has a higher performance level with respect to one function, while having a lower performance level with respect to another function. This enables models to reflect a complex range of responses from an NP battery.

A probability distribution on the states is used to represent belief about which state best describes the cognitive capabilities of a subject. Bayes' rule is used to obtain updated posterior probabilities of state membership once responses to measures are observed. This allows for a systematic manner in which the information obtained from observing multiple measures can be combined for statistical classification. Two response distributions are estimated per NP measure, one representing the response tendencies of subjects who perform at a relatively high level on all functions associated with the particular measure and another for those subjects who do not. These distributions are used to weigh the relative likelihood of an observed response indicating that a subject has the associated higher level functioning. The approach of estimating two response distributions per NP measure is parsimonious in that there is no need to estimate response distribution parameters that are specific to each profile; rather, response distribution parameters are shared either by profiles that have the associated functioning levels, or those that do not. Ideally, one state in the model will have a probability close to 1 for each individual, while other states will have probabilities near 0, indicating that a subject's cognitive profile is known with near certainty. As long as models are correctly specified, this near certainty will indeed be obtained given a sufficient amount of testing [7]. Once classification is completed, subjects who share a cognitive profile can be aggregated, and observed rates of conversion to AD between the resulting subgroups can be compared.

In our study, we used NP data collected by the Alzheimer's Disease Neuroimaging Initiative (ADNI) [9] to evaluate the usefulness of poset models to identify specific cognitive phenotypes associated with conversion from MCI to AD [7,8,10,11]. We hypothesized that poset modeling would generate interpretable and sufficiently detailed cognitive phenotypes with clearly differentiated rates of conversion from MCI to AD. Given its established importance in progression risk for AD, Apolipoprotein E (APOE) e4 status was also taken into account [12,13].

## Materials and methods

### Study sample

MCI subjects enrolled in the ADNI (*n *= 389) were included in the classification analysis if their scores on the selected NP battery were available at both baseline and at 24 months. The sample was 64.5% male and 93.1% Caucasian. About 3.8% were African American, 2.8% were Asian, and 0.3% were American Indian or Alaskan Native. Mean age was 74.8 years (SD = 7.5) and mean length of education was 15.7 years (SD = 3.0).

### Modeling and classification approach

We used neuropsychologist expert opinion (JJ and HYT) to map the relationship between selected ADNI NP measures (ADAS delayed recall and word recognition subscales and number cancellation; auditory verbal learning test (AVLT) Trial 6 and List B; Boston naming test; category fluency; trail making Test A and Test B, and Wechsler adult intelligence scale-revised (WAIS-R) digit symbol substitution) and the cognitive functions required to perform them (episodic memory at four different levels, word fluency, cognitive flexibility, perceptual motor speed, and attention). See Table 1 for the listing of these specifications. Measures were selected based on the types of functions they tested and retained based on statistical criteria such as discriminatory properties and correspondence with model fit. Given the reliance on expert opinion, data-analytic validation is important. Statistical details on how this validation was performed are provided in Additional file 1 (Appendix).

**Table 1:** Cognitive functions and how they are tapped into by selected ADNI neuropsychology measures, as specified according to expert opinion

ADNI measure	Cognitive function						
	
	Episodic memory level 1	Episodic memory level 2	Episodic memory level 3	Word fluency	Selective attention	Cognitive flexibility	Perceptualmotor speed
ADAS-delayed recall subscale	X	X	X		X		
ADAS word recognition subscale	X				X		
AVLT-Trial 6	X	X	X		X		
AVLT-List B	X	X			X		
Boston naming test				X	X		
Category fluency^*^				X	X	X	
ADAS-number cancellation					X		
Trail making test A					X		X
Trail making test B					X	X	X
WAIS-R digit symbol substitution					X		X

ADAS, Alzheimer's Disease Cooperative Study; ADNI, Alzheimer's Disease Neuroimaging Initiative; AVLT, adult verbal learning test; WAIS-R, Wechsler adult intelligence scale-revised; ^*^average of vegetable and animal categories.

To acknowledge the key role of episodic memory impairment as an early symptom in AD and to better represent the varying levels of episodic memory required across the measures, we identified four levels of episodic memory proficiency (levels 0 through 3). These levels are ordered in terms of episodic memory demand, with level the highest, and level 0 the lowest. Level 3 corresponds to relatively longer-term delayed recall with distractors, while level 2 and level 1, respectively, relate to shorter and shorter recall durations involved in AVLT List B and immediate recall. A subject at level 0 cannot perform well at any of the other levels. A brief summary of the practical interpretation of the episodic memory levels to be used here is given in Table 2.

**Table 2:** Description of episodic memory functioning levels used in the model

Level	Description
Level 3	Relatively longer-term delayed recall with distractors
Level 2	Relatively shorter recall durations, such as involved in AVLT List B
Level 1	Immediate recall, word recognition
Level 0	Cannot perform well at any of the above levels

AVLT, adult verbal learning test

Through the assumed hierarchical ordering between levels of episodic memory, note that the delayed recall measures that involve level 3 also can provide information on functioning with levels 1 and 2. High functioning at level 3 implies high functioning at levels 1 and 2 as well. Also, poor performance on word recognition, which is associated with level 1, not only provides evidence that level 1 is at a low level, but that functioning at levels 2 and 3 should be low as well. Hence, even though associations of measures involving episodic memory can be at different levels, they still can inform other levels of episodic memory functioning, affording some degree of replication.

### Poset model generation

A poset model was generated based on the functions associated with each of the ADNI NP measures. In the cognitive profiles associated to states in the model, performance levels for a function were denoted either as relatively high, or relatively low, in relation to the MCI and early AD sample. See Table 3 for a complete list of profiles that can be distinguished by the given set of NP measures that were analyzed.

**Table 3:** Poset states and their corresponding profiles of cognitive functioning relative to mild cognititve impairment and Alzheimer's disease

Poset state	Cognitive function						
	
	Selective attention	Episodic memory level 1	Episodic memory level 2	Episodic memory level 3	Word fluency	Cognitive flexibility	Perceptual motor speed
1	X	X	X	X	X	X	X
2	X	X	X	X	X	X	
3	X	X	X	X	X		X
4	X	X	X	X	X		
5	X	X	X	X		X	X
6	X	X	X	X			X
7	X	X	X	X		*	X
8	X	X	X		X	X	X
9	X	X	X		X	X	
10	X	X	X		X		X
11	X	X	X		X		
12	X	X	X			X	X
13	X	X			X		X
14	X	X	X			^*^	
15	X	X			X	X	X
16	X	X			X	X	
17	X	X			X		X
18	X	X			X		
19	X	X				X	X
20	X	X					X
21	X	X				^*^	
22	X				X	X	X
23	X				X	X	
24	X				X		X
25	X				X		
26	X					X	X
27	X						X
28	X					^*^	
29							

X indicates relatively high functioning; ^*^undetermined..

**Figure 1:**
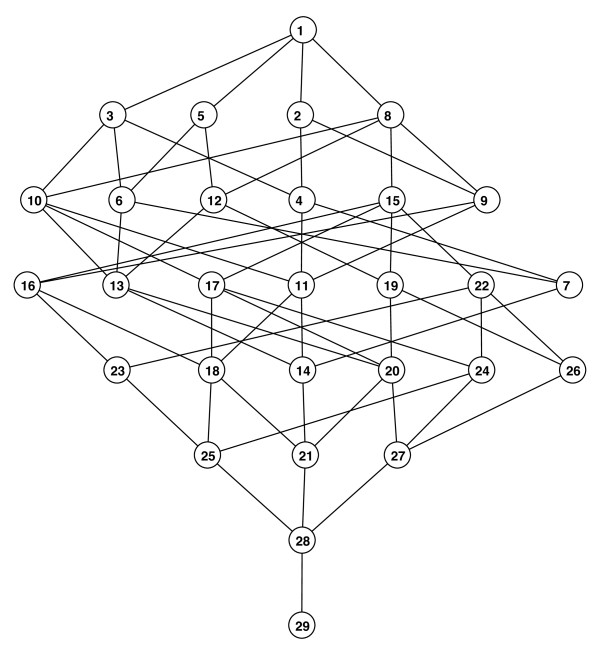
**Partially ordered set (poset) model for cognitive functioning of mild cognifitive impairment and early Alzheimer's disease subjects**.

The resultant poset model comprised 29 states. These states (and associated cognitive profiles) represent the profiles that can be distinguished from the NP battery, and were identified algorithmically from the expert-derived specifications in Table 1[14]. Table 3 shows how the cognitive functions we examined are distributed across states to create distinct cognitive profiles. The corresponding partial ordering of these profiles is graphically depicted in Figure 1 as a Hasse diagram. Note that state 1 is the highest state in the poset, as it has the highest level of functioning for all five cognitive functions. State 29 is the lowest state, since all its functioning for all five functions is at the lowest level. For the in-between states, there exists at least one function for which performance is not at a relatively high level. In the diagram, connected lines between states indicate direct ordering, with the higher of the states being greater than the lower one.

In our analysis, a uniform prior probability value of 1/29 was assigned to each state for each subject to indicate prior belief about which profile would fit a given subject. We then estimated two response distributions for each NP measure as described above. These distributions were then used to weigh the relative likelihood that an observed response indicated that a subject had the associated higher level functioning.

### Response distributions

Responses from both the MCI subjects and also early AD subjects (an additional 174 such subjects) were included in the estimation, to allow for a range of values. Given the apparent non-normality of response data, nonparametric approaches to response distribution estimation were adopted [10,11,15-17] (see Additional file 1).

### Grouping of profiles and classified subjects

The ordered relationships between states arise when identifying subgroups with shared functioning levels for a function. For instance, the subgroup of states that have high performance level for episodic memory level 2 are all the states greater than or equal to state 14. Precisely, this would be state 1 through 12, and state 14. The complement of this subgroup (all states not greater than or equal to state 14) would thus comprise the states with lower performance level. Once subgroups such as this have been identified and classification conducted, the probability that a subject has a particular performance level for a function can be computed by summing the posterior probabilities of membership of each of the states in the subgroup. These probabilities are used as a basis for cutoff values in function-related groupings, which are then compared statistically in terms of proportion of AD conversions from MCI. All reported *P*-values are two-sided.

We treated cognitive flexibility slightly differently from other NP functions, due to confounding of its functioning status in classification under certain profiles, specifically for states 7, 14, 21, and 28. Confounded profiles arise due to limitations of the NP battery to distinguish all possible profiles. Profiles with confounding give conflicting information about certain functions, but probabilities for a subject being at certain functioning levels can still be obtained by weighting the information provided across a set of confounded profiles (see Additional file 1 for more details).

### Model validation

Briefly, model fit appears to be good. Response distribution estimates for all measures correspond to the assumed order structure, in that those subjects expected by the model to score well actually tended to do so, and those not expected to score well tended not to do so. Moreover, classification was fairly decisive, especially given the limited number of NP measures employed. Observed responses to the measures were thus consistent with the model specifications. See Figures S1 through S6 and Table S1 in Additional file 1.

## Results and discussion

We explored different cognitive groupings within MCI subjects, to assess conversion rates to AD at two years post baseline. Not surprisingly, episodic memory is the cognitive function that appears to be most significantly related to future conversion. Still, the different performance levels of episodic memory in our model appear to have varying degrees and ways of association with conversion outcomes. For instance, as seen in Table 4, level 2 episodic memory performance levels influence the rate of conversion among those with MCI. In subjects for whom level 2 episodic memory functioning is low (in other words, below the cutoff probability value of less than 0.275), 41 out of 67 (61.2%±11.7%, 95% CI) converted to AD within two years, which is much higher than the overall MCI to AD conversion rate in this sample of 101 out of 268 (37.7%±5.8%, 95% CI). Those with relatively lower performance in level 2 of episodic memory significantly differ in conversion rates compared with those with higher performance, regardless of whether or not the APOE e4 allele is present. The *P*-value for Fisher's exact test is 0.000 for a two-sided test of no association between conversion and having relatively low episodic memory level 2 functioning.

**Table 4:** Relationship between episodic memory level 2 functioning and conversion to Alzheimer's disease (AD) over a two-year period

Converted to AD	Low episodic memory level 2 function	Not low episodic memory level 2 function	Total
**Yes**	61.2% (41)	29.9% (60)	37.7% (101)
**No**	38.8% (26)	70.1% (141)	62.3% (167)
**Total**	100% (67)	100% (201)	100% (268)

Results are presented as the percent (number) of patients. Fisher's exact test two-sided *P*-value = 0.000 for a test of no association; positive predictive value = 0.612; negative predictive value = 0.701.

Other functions where relatively lower functioning at baseline may indicate higher risk for conversion from MCI to AD are perceptual motor speed and cognitive flexibility. For perceptual motor speed, using a cutoff probability value of 0.40 to delineate a lower functioning subgroup, and considering subjects with at least one APOE4 allele, 23 of 35 (65.7%±15.7%, 95% CI) of MCI subjects with relatively low functioning convert to AD within 24 months. On the other hand, only 14 of 35 MCI subjects with relatively low perceptual motor speed and without an APOE4 allele convert (40%±16.2%, 95% CI). Further, for cognitive flexibility, using a cutoff probability value of 0.30 to delineate a lower functioning subgroup, 28 of 48 (58.3%±13.9%, 95% CI) of MCI subjects with relative low baseline functioning convert to AD within 24 months. In this case, the *P*-value for Fisher's exact test of no association is 0.007.

Interestingly, we conversely found much lower rates of conversion among certain cognitive profiles. In particular, only four out of forty-one (9.8%±9.1%, 95% CI) MCI subjects with no APOE e4 alleles and relatively high level-3 episodic memory functioning (cutoff probability value greater than 0.80) convert in two years. This rate appears to be lower than for subjects with no APOE e4 allele but without high level-3 episodic memory functioning (*P*-value = 0.001, Fisher's exact test of no association).

### Amnestic multidomain MCI identified with poset models

An advantage of the poset approach is the ability to provide classification to profiles that address a range of functions. Subgroups, such as amnestic multidomain MCI, can be characterized more precisely by identifying specific functions that are relatively impaired along with episodic memory.

In our sample, subjects with relatively low functioning on episodic memory level 2 (probability of being at high level at level 2 being 0.275 or less) and perception speed (probability cutoff value of 0.40 or less) were identified. As shown in Table 5, among subjects with at least one APOE e4 allele and with relatively low levels of both episodic memory and perception speed, 16 out of 19 converted (84.2%±16.4%, 95% CI). Considering only subjects with lower level-2 episodic memory functioning, those who additionally have lower functioning in perceptual motor speed appear even more likely to convert (*P*-value = 0.013, Fisher's exact test of no association). In contrast, additionally having lower functioning with cognitive flexibility did not appear to significantly increase risk for conversion. Among subjects with lower episodic memory level 2 functioning, observed percentages of conversion with and without lower cognitive flexibility were 66.7% versus 57.5% (18 out of 27 versus 23 out of 40).

**Table 5:** Relationship between episodic memory level 2 and perceptual motor speed functioning and conversion to AD over a two-year period by APOE4 allele status

Converted to AD	Low episodic memory function and no APOE4 allele^a^			Low episodic memory function and at least one APOE4 allele^b^		
		
	Low perceptual motor speed function	Not low perceptual motor speed function	Total	Low perceptual motor speed function	Not low perceptual motor speed Function	Total
**Yes**	63.6% (7)	54.5% (6)	59.1% (13)	84.2% (16)	37.8% 17)	61.2% (18)
**No**	36.4% (4)	45.5% (5)	40.9% (9)	15.8% (3)	62.2% (28)	38.8% (10)
**Total**	100% (11)	100% (11)	100% (22)	100% (19)	100% (13)	100% (28)

Results are presented as the percent (number) of patients.^a^Fisher's exact test two-sided *P*-value = 1.00 for a test of no association. ^b^Fisher's exact test two-sided *P*-value = 0.013 for a test of no association. AD, Alzheimer's disease; APOE, Apolipoprotein.

### Cognitive change before conversion

**Figures 2:**
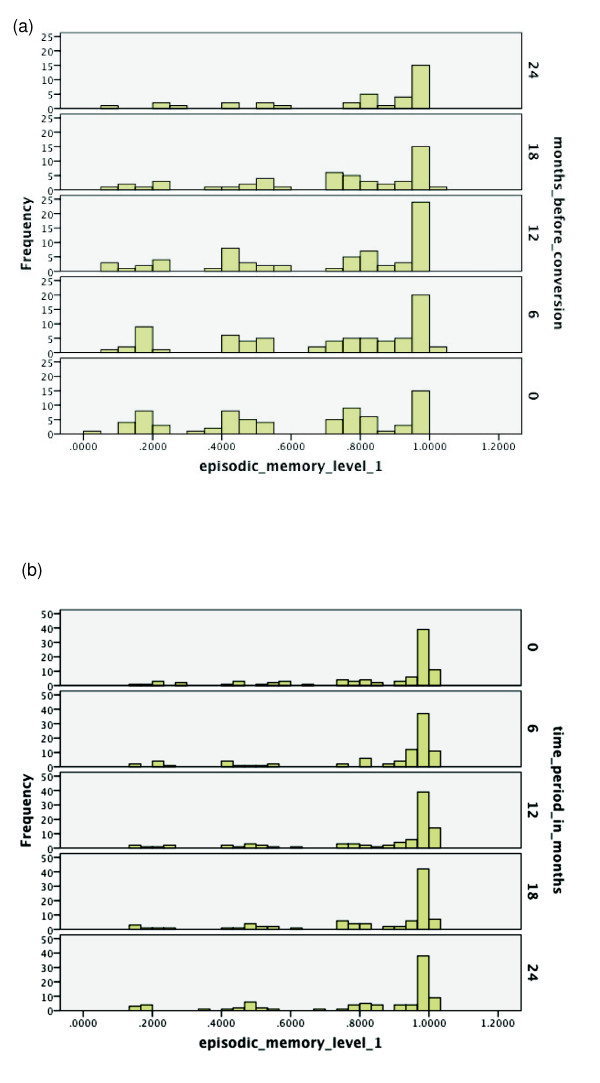
**Probabilities of functioning over time among mild cognitive impairment (MCI) converters to Alzheimer's disease (AD) versus MCI non-converters**. (**a**) Probability of relatively high functioning with episodic memory level 1 from 24 to 0 months before conversion to AD among MCI subjects. Respectively, *n *= 75, 75, 68, 51, and 36 for 0, 6, 12, 18 and 24 months before conversion. (**b**) Probability of relatively high functioning with episodic memory level 1 from baseline (0 months) to 24 months among MCI subjects who did not convert within 36 months; *n *= 90.

**Figure 3:**
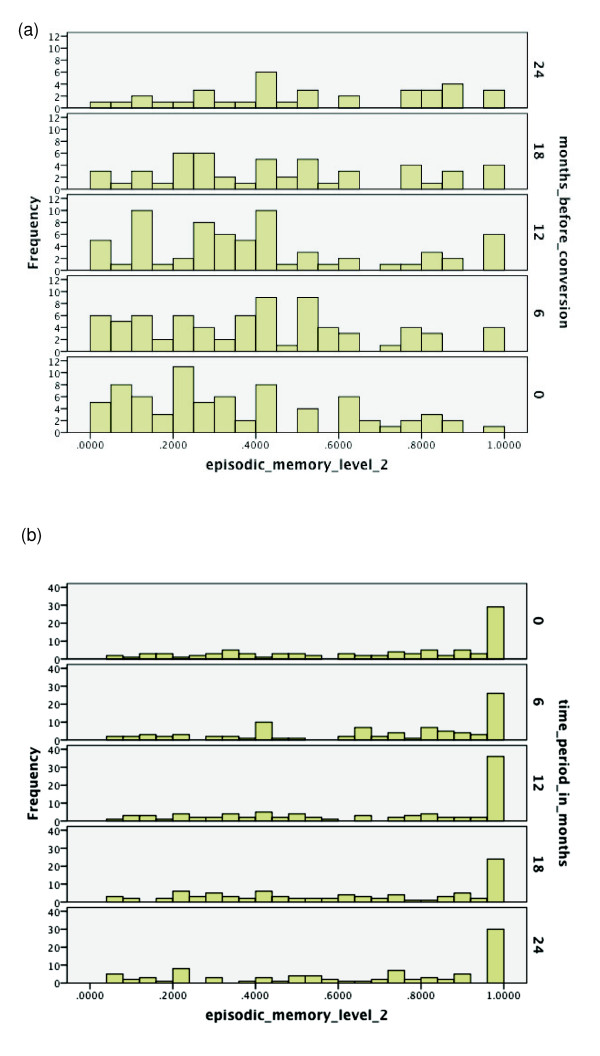
**Probabilities of functioning over time among mild cognitive impairment (MCI) converters to Alzheimer's disease (AD) versus MCI non-converters**. (**a**) Probability of relatively high functioning with episodic memory level 2 from 0 to 24 months before conversion to AD among MCI subjects. (**b**) Probability of relatively high functioning with episodic memory level 2 from baseline to 24 months among MCI subjects who did not convert within 36 months.

**Figure 4:**
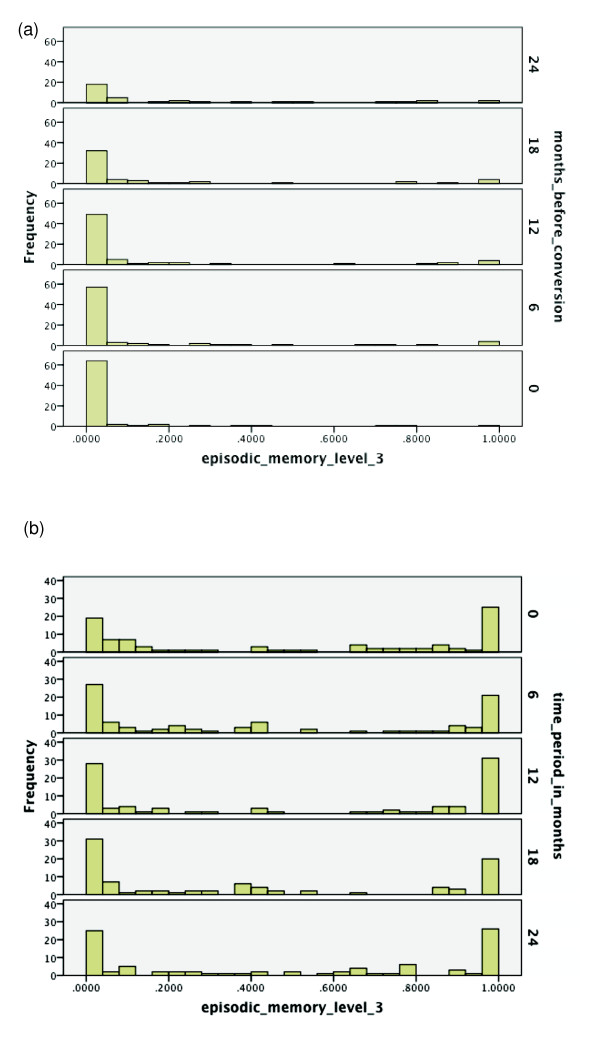
**Probabilities of functioning over time among mild cognitive impairment (MCI) converters to Alzheimer's disease (AD) versus MCI non-converters**. (**a**) Probability of relatively high functioning with episodic memory level 3 from 24 to 0 months before conversion to AD among MCI subjects. (**b**) Probability of relatively high functioning with episodic memory level 3 from baseline to 24 months among MCI subjects who did not convert within 36 months.

**Figure 5:**
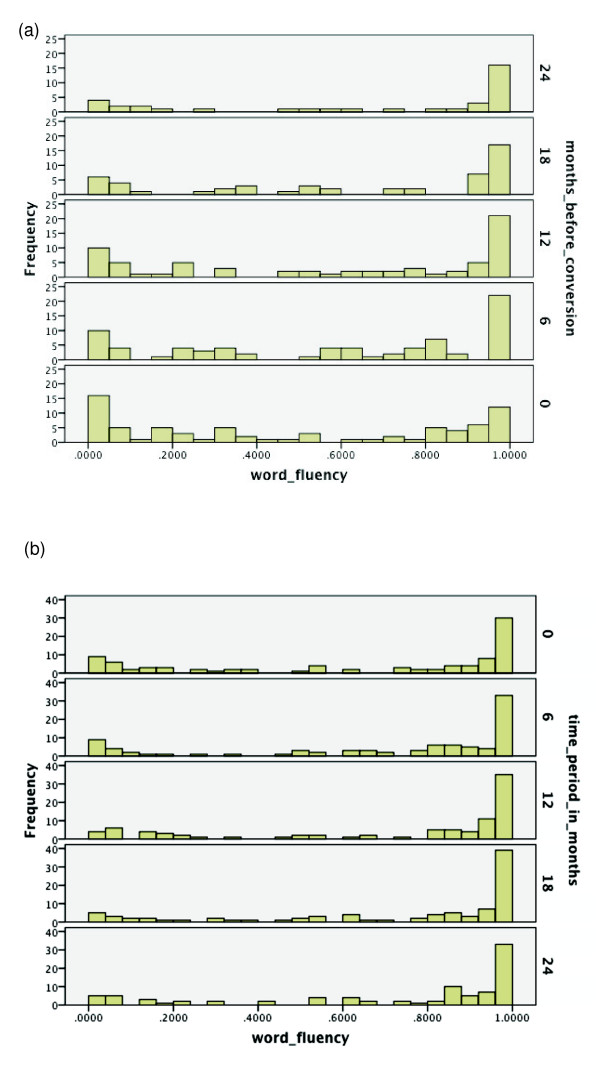
**Probabilities of functioning over time among mild cognitive impairment (MCI) converters to Alzheimer's disease (AD) versus MCI non-converters**. (**a**) Probability of relatively high functioning with word fluency from 24 to 0 months before conversion to AD among MCI subjects. (**b**) Probability of relatively high functioning with word fluency from baseline to 24 months among MCI subjects who did not convert within 36 months.

**Figure 6:**
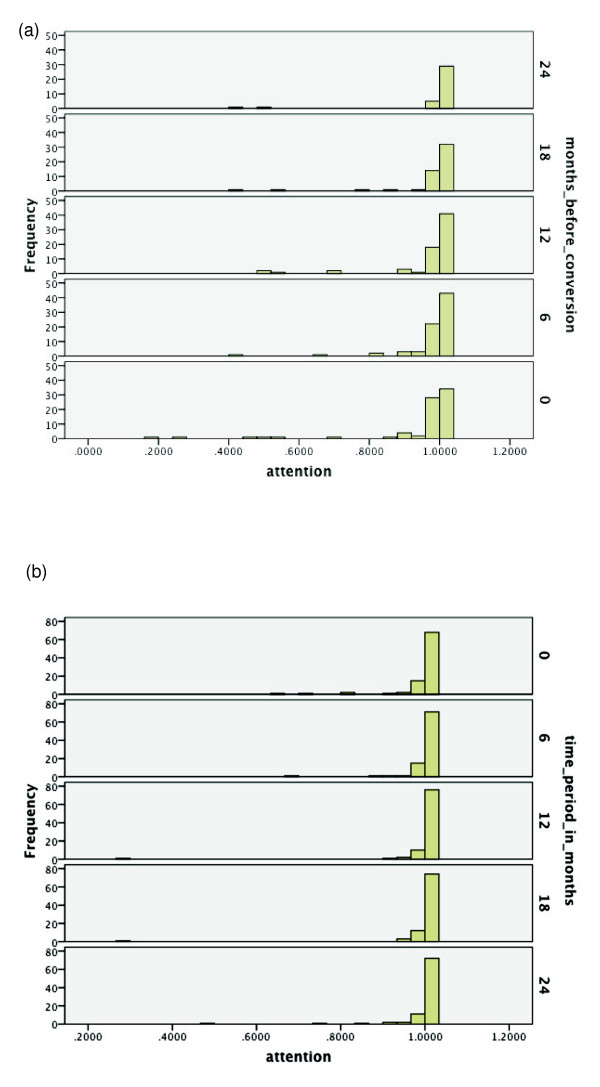
**Probabilities of functioning over time among mild cognitive impairment (MCI) converters to Alzheimer's disease (AD) versus MCI non-converters**. **(a**) Probability of relatively high functioning with attention/sustained vigilance from 24 to 0 months before conversion to AD among MCI subjects. (**b**) Probability of relatively high functioning with attention/sustained vigilance from baseline to 24 months among MCI subjects who did not convert within 36 months.

**Figure 7:**
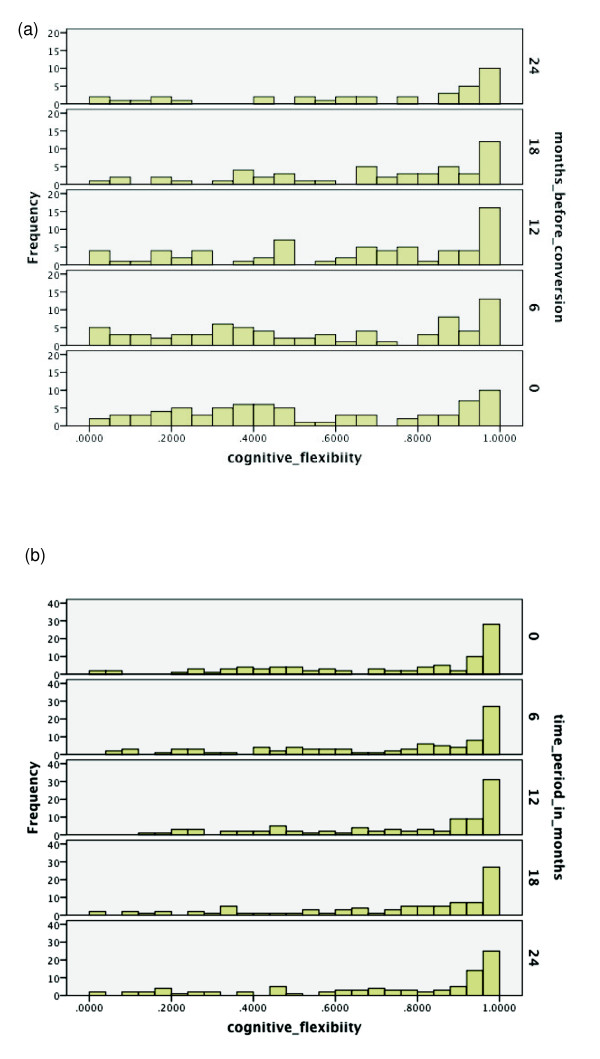
**Probabilities of functioning over time among mild cognitive impairment (MCI) converters to Alzheimer's disease (AD) versus MCI non-converters**. (**a**) Probability of relatively high functioning with cognitive flexibility from 24 to 0 months before conversion to AD among MCI subjects. (**b**) Probability of relatively high functioning with cognitive flexibility from baseline to 24 months among MCI subjects who did not convert within 36 months.

**Figure 8:**
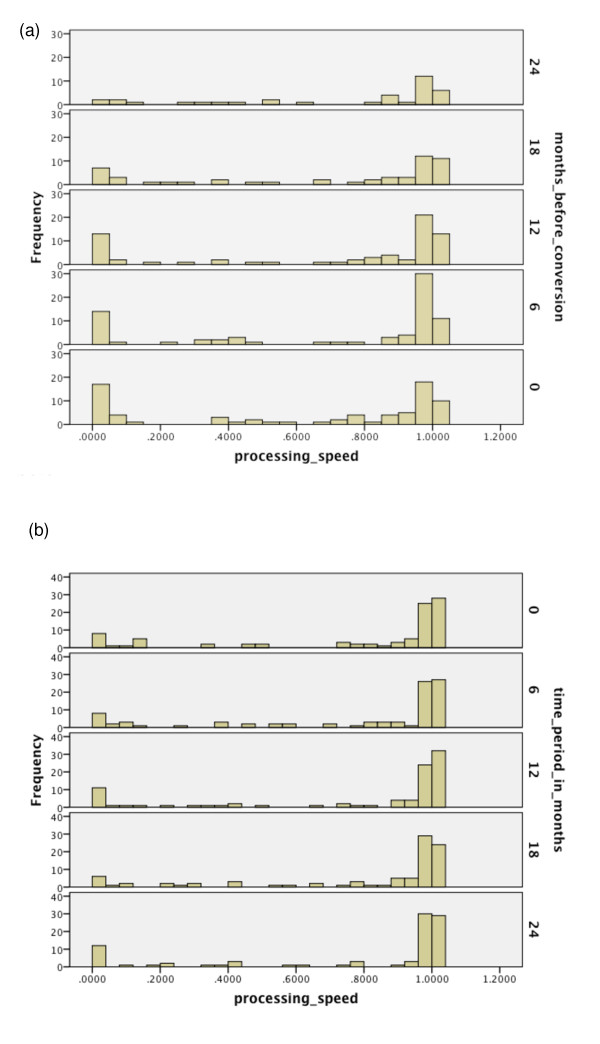
**Probabilities of functioning over time among mild cognitive impairment (MCI) converters to Alzheimer's disease (AD) versus MCI non-converters**. (**a**) Probability of relatively high functioning with perceptual motor speed from 24 to 0 months before conversion to AD among MCI subjects. (**b**) Probability of relatively high functioning with perceptual motor speed from baseline to 24 months among MCI subjects who did not convert within 36 months.

Over the range of cognitive functions, histograms were generated for the respective probabilities of converters having relatively high functioning over the 24 months preceding conversion. Analogous histograms were generated for non-converters over a time period of the same duration starting from baseline. See Figures 2, 3, 4, 5, 6, 7, 8. Note that for non-converters, only those subjects who did not convert over the full 36-month period of the study were included in the plots. By comparing corresponding plots between converters and non-converters, it becomes clearer how the above findings of heterogeneity in conversion outcomes by cognitive profile arose.

For Figures 2, 3, 4, which correspond to the episodic memory levels, note that relative to level 2, level 1 does not see the same amount of decline over 24 months for converters, as reflected by a shift to lower probability values. Hence, the discrepancy between the histograms in Figures 2a versus 2b over time between converters and non-converters is not very strong. On the other hand, in Figures 3a and 3b, it is clear that for converters, there is quite a bit of decline in level 2 values during this time period, while non-converters appear stable. This makes level 2 attractive for discrimination and prediction over this duration. In Figures 4a and 4b, note that level 3 functioning is low among almost all converters preceding conversion. However, lower functioning for this more difficult level also is common for non-converters, lessening the discriminatory properties of level 3. Still, there is a sizeable proportion of non-converters retaining high probability values for level 3, which allows for cognitively-based identification of a very low-risk group.

In Figures 5a and 5b, it appears that there may be decline in word fluency values for a relatively small subset of subjects as they near conversion, but many converters also appear to retain high functioning. For non-converters, some subjects have lower functioning as well. Hence, word fluency does not appear useful for predicting conversion within a two-year period. In Figures 6a and 6b, note that there may be some slight decline in attention/sustained vigilance for converters, but almost all subjects still retain high probability values for being at a high level of attention functioning.

In Figures 7a and 7b, there is definitely a fair amount of decline in values for cognitive flexibility among converters, although it is also not as pronounced as for episodic memory level 2. Also, some converters retain high functioning. Most non-converters retain high functioning over the duration. Hence, cognitive flexibility can be useful for discriminating future conversion outcomes, but does not appear as informative as episodic memory level 2. Finally, in Figures 8a and 8b, for perceptual motor speed, note that there appears to be a subset of converters for whom perceptual motor speed becomes more impaired. While there are non-converters who also have low probability values, this number is outweighed by the converters over the duration. Moreover, as Table 5 indicates, these converters are likely to also be relatively more impaired with episodic memory level 2 than the non-converters. This allows us to identify this particular combination of lower level functioning as being specifically associated with high risk for conversion.

### Multivariate prediction using logistic regression models

A multivariate model was fit that recognized the above findings, and included other variables such as gender, age, and educational level. The presence of an APOE4 allele was viewed as a binary variable, as well as whether or not a subject had attended college. Also, probability values for performing at a relatively high level for episodic memory level 2, cognitive flexibility, and perceptual motor speed were viewed as continuous explanatory variables. After an initial fit of a full model, gender, age, and educational level were clearly not significant predictors in the model (respective *P*-values were 0.96, 0.65, and 0.81; Wald's test). Using goodness-of-fit tests based on the test statistic of -2 times the difference in log-likelihood values to compare nested models, it appears the best fit is when episodic memory level 2, perceptual motor speed, and APOE4 status are included in the model. Results are given in Table 6. Note that episodic memory level 2, perceptual motor speed, and APOE4 status all are significant predictors (*P*-value <0.05; Wald's test). When cognitive flexibility is included with these variables, it is not significant (*P*-value = 0.26).

**Table 6:** Multivariate logistic regression model with outcome as conversion to Alzheimer's disease (AD) from mild cognitive impairment (MCI) within 24 months from baseline

Explanatory variables	B	Wald test *P*-value	Exp (B)
**APOE4**	0.623	0.024	0.536
**Episodic memory level 2**	-1.445	0.001	0.236
**Perceptual motor speed**	-0.926	0.009	0.396
**Intercept**	1.193	0.000	3.298

Dependent variable value of 1 indicates that a subject has converted to AD from MCI within 24 months of baseline. Binary variable Apolipoprotein (APOE)4 = 1 indicates that the subject has at least one APOE4 allele and higher risk for conversion. Larger probability values of relatively high performance indicate lower risk for conversion.

**Figure 9:**
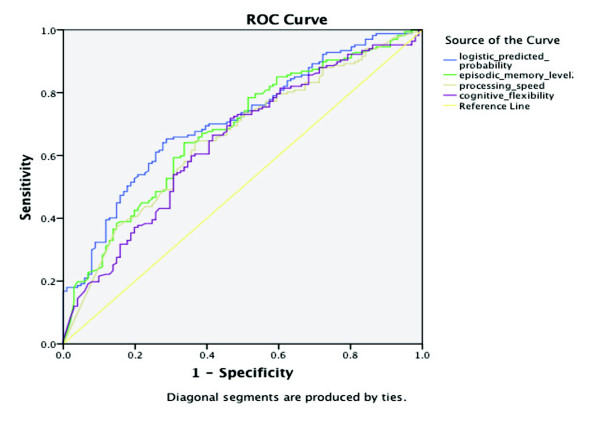
**Area under the curve (AUC)**. Multivariate logistic model probability values for conversion = 0.710, episodic memory level 2 probability of high functioning = 0.678, perceptual motor speed probability of high functioning = 0.655, cognitive flexibility probability of high functioning = 0.644.

Using the model-based estimated probabilities of converting to AD as a predictor, and for instance, using a cutoff value of 0.55 or higher, classification accuracy is 66.8%, with positive predictive value of 61.5% (32 out of 52). Figure 9 displays receiver operating characteristic (ROC) curves for these logistic regression probabilities, as well as for the probabilities for episodic memory level 2, perceptual motor speed, and cognitive flexibility. Values for the area under the curve (AUC) are 0.710, 0.678, 0.655, and 0.644, respectively. Using a multivariate approach can thus apparently improve prediction. However, compared to relying upon episodic memory level 2 alone without the need for APOE4 status information, which would have practical appeal, the difference does not appear to be striking.

### Other prediction approaches

A comprehensive review of research efforts using ADNI data is given in Weiner *et al*. (2012) [18]. This includes a description of work on prediction of MCI to AD conversion. Taking advantage of the richness of the ADNI data, prediction models have been developed based on a range of various imaging, cerebrospinal fluid, and genetic biomarkers, as well as cognition. Our prediction results appear to be comparable to non-cognitively oriented methods that rely on baseline data [19,20]. Advantages of a cognitive testing approach include non-invasiveness and cost, especially if focused and efficient NP batteries can be designed, and computer-based adaptive testing adopted in the future.

Other cognitive testing-based approaches to prediction include Tabert *et al*. (2006) [21] and Fleisher *et al*. (2007) [22]. Their prediction models depend directly on NP measurement scores, which in general may be difficult to interpret in terms of identifying which cognitive functions may be the source of poor scores. We believe that the results presented here add to these works, such as through a more specific consideration of multidomain MCI. Poset-based methods also provided insight into the course of cognitive change in MCI, by indicating how specific functions are affected over time. The findings depicted in Figure 4 allow for insight into the heterogeneity in cognitive progressions that arise among MCI, and thus help in identifying profiles of high risk.

## Conclusions

Our results suggest the utility of the poset-based approach in uncovering heterogeneity in risk for conversion from MCI to AD by generating subgroups tied to specific cognitive functions. Duration of 24 months from baseline measurement was considered. Among the cognitive functions evaluated, episodic memory was mostly strongly linked to conversion from MCI to AD. This confirms similar findings in Tierney *et al*. (2005) [23], Tabert *et al*. (2006) [21], Blacker *et al*. (2007) [24], and Landau *et al*. (2010) [18]. We did find that cognitive flexibility and perceptual motor speed also is associated with conversion, as certain subjects are apparently affected in these domains during the 24 months preceding conversion. Conversely, MCI subjects with relatively less episodic memory impairment were observed to convert at a much lower rate. The importance of the APOE e4 allele in affecting risk for conversion is also clear [18,19].

More precisely, it appears that in our model certain levels of episodic memory functioning are more discriminatory than others in terms of identifying MCI subjects at especially high risk for conversion. In particular, level-2 episodic memory functioning, more so than level-3, appears to best identify risk for conversion. It appears that delayed recall with distractors may be too difficult a task to discriminate risk very well. On the other hand, for those that can perform well at level 3 relative to others with MCI, risk of conversion is much lower, as illustrated in Table 5.

Poset modeling also appears to be helpful in further clarifying the notion of multidomain MCI. Our analyses suggest that perceptual motor speed functioning may have a stronger link to subsequent risk of AD progression than other cognitive functions when considered in conjunction with relatively reduced function of level-2 episodic memory. On the other hand, additional impairment with cognitive flexibility does not appear to increase risk beyond that due to episodic memory impairment. These results suggest that having relatively lower functioning across multiple functions can indeed increase risk for AD, but, that it may matter which of the functions are impaired. Because corresponding samples become smaller as specific combinations of deficits are analyzed, however, these analyses must be viewed as preliminary.

A possible concern is that the ranges of performance levels for functions in the poset model are limited, particularly when a functioning level is either high or low. However, it should be kept in mind that there are also limited NP response data available, due to the time-consuming and burdensome nature of NP measurement. Hence, there are statistical limitations to the granularity of information on functioning that can be assessed accurately. Our model was shown to be feasible, while still being able to provide discriminating information relating to AD conversion. Another limitation is the scope of inference that can be made from the ADNI sample, which was overwhelmingly Caucasian, and imbalanced towards males. Certainly, these findings should be validated in other datasets.

### Future directions

Since the poset model allows for precise and detailed cognitive profiles, this approach can be used in conjunction with imaging and genetic studies. As an example, a cluster analysis approach has recently been applied to characterize MCI subgroups by cognitive characteristics [25]. While white matter lesion burden was found to differ by these groupings, in general, cognitive subtypes resulting from cluster analysis can at times be more difficult to interpret than profiles generated with poset models. Also, note the persistent heterogeneity of conversion outcomes among MCI subjects even within the cognitively homogeneous subgroups generated here. These subgroups could be an interesting basis for functional magnetic resonance imaging (fMRI) studies of cognitive reserve, which may improve understanding of how this heterogeneity arises [26,27]. Poset modeling may also be useful in clinical trial design, as enriched sampling from subgroups of MCI subjects that have high positive predictive value of conversion could reduce sample size requirements.

Finally, poset models can serve as a basis for adaptive NP testing, with NP measures being selected for administration dynamically, based on the responses that have already been observed [7,8]. As demonstrated, an attractive feature of poset models is that since they are comprised of discrete states, accurate statistical classification can be conducted with relatively few measures. Adaptive tests can further reduce subject burden and allow for more focused and efficient testing, which in turn would enhance the appeal of cognitive testing for prediction.

## Abbreviations

AD: Alzheimer's disease; ADAS-Cog: Alzheimer's disease assessment scale-cognitive; ADNI: Alzheimer's Disease Neuroimaging Initiative; APOE: Apolipoprotein E; AUC: area under the curve; AVLT: auditory verbal learning test; fMRI, functional magnetic resonance imaging; MCI: mild cognitive impairment; MMSE: mini-mental status exam; NP: neuropsychological; poset: partially ordered set; ROC; receiver operator characteristic; WAIS-R: Wechsler adult intelligence scale-revised.

## Competing interests

Dr Tatsuoka was funded in part by AstraZeneca Pharmaceuticals to do this research, and has written a related patent. Drs Tseng, Varadi, Smyth and Yamada have no conflicts of interest to report. Dr Jaeger was Director, Global Medicines Development, Neuroscience, AstraZeneca Pharmaceuticals during the funding of this research, and is now with CogState, Inc. Dr Smith was a consultant for Anavex Life Sciences Corporation, Medivation, Eisai, Glaxo Welcome Kline, and Neurotez; he owns stock options in Neurotez, Aria, Panancea, and Curaxis Pharmaceuticals. Dr Lerner receives research support from Forest Labs, Pfizer, Medivation, Baxter Labs, and Avid Pharmaceuticals.

## Authors' contributions

CT developed the statistical methods, conducted statistical analyses, and helped prepare the manuscript; HYT conducted analyses; JJ helped frame and conduct the analyses; FV programmed the software, helped in developing the statistical methods, and helped in the analysis; MS helped in the analyses; TY conducted analyses; KS helped in the analyses and prepared the manuscript; AL helped frame and conduct the analyses, and prepare the manuscript. All authors have read and approved the manuscript for publication. Data used in the preparation of this article were obtained from the ADNI database [28]. As such, the investigators within the ADNI contributed to the design and implementation of ADNI and/or provided data but did not participate in analysis or writing of this report. ADNI investigators include a complete listing available at [29].

## Supplementary Material

Additional file 1 **Appendix: Statistical framework for data analysis and model validation**. This file contains statistical details relating to the poset modeling, including parameter estimates and classification summaries. It also describes how model validation was conducted. Click here for file
