# Supplementary material for: Modeling the heterogeneity in risk of progression to Alzheimer's disease across cognitive profiles in mild cognitive impairment
Source: Alzheimers Res Ther. 2013 Mar 6;5(2):14. doi: 10.1186/alzrt168 (PMC3707057; doi:10.1186/alzrt168)
Supplement: Additional file 1 — Appendix: Statistical framework for data analysis and model validation. This file contains statistical details relating to the poset modeling, including parameter estimates and classification summaries. It also describes how model validation was conducted. [file alzrt168-S1.PDF]

Modeling the heterogeneity in risk of progression to Alzheimer's disease across cognitive profiles in mild cognitive impairment

APPENDIX: Statistical framework for data analysis and model validation

The statistical framework follows as in Tatsuoka (2002) [1], where further details are given. Also see Jaeger et al. (2006a,b) [2], [3]. Briefly, a Bayesian approach to classifying subjects to a state is adopted, so that prior probabilities of state membership are assigned for each test subject. As mentioned, a uniform, non-informative prior probability was assigned to each of the profiles, with each state in the poset model being viewed as equally likely to be the true one prior to updating the probabilities of state membership through observed test scores. Data-analytic validation of model fit and of the cognitive specifications associating functions with measures is essential to providing reliable and accurate results, as the cognitive processes underlying the assessment responses are latent and complex. The data-analytic tools for assessing model fit and validating cognitive specifications involve analyzing NP test distribution estimates and patterns in the classification results are given in Tatsuoka (2002) [1]. We follow that approach here. A table and figures are given after the references.

Estimation of the distributions of responses for the measures depending on a subject's profile, and implications for model validation: For each measure, one of two types of response models was estimated using Bayesian procedures and Markov Chain Monte Carlo simulation methods. One is the multinomial distribution, with estimation following as in Tatsuoka (2002) [1] and Jaeger et al. (2006a,b) [2], [3]. Uniformly distributed and non-informative Dirichlet conjugate priors were employed. The other response models were normal mixtures models. These models allow for the fitting of

complex and non-parametric distributions. Non-informative priors were selected in the estimation of the normal mixtures as well, as detailed in Ishwaran and James (2002) [4] and Tatsuoka et al. [5]. Ten thousand iterations using Gibbs sampling were run after a burn-in period, with stationary convergence attainment assessed as in Geweke (1992) [6]. Sensitivity analysis for priors was conducted, in that some modifications to prior specifications were made, to see if classification results differ. As MCI and early AD subjects in ADNI combined to a relatively large baseline sample size, estimation results were not sensitive to moderate adjustment of prior specifications.

Consider the NP test response distribution estimates in Table S1. This table displays, for 8 out of the 10 NP measures, the posterior means and standard deviations of the multinomial probability parameters. Trails A and B are not included, as normal mixture model were used for those measures. Test performance scoring ranges for the included measures are divided into quartiles, and respective multinomial distributions were estimated. The decision to use four response categories demarcated by quartiles was based on good observed classification performance and the preservation of expected order-based relationships between respective estimated distributions for measures. Overall, the differences in the estimated probability values between the two response distributions for each measure, especially for the first and fourth quartiles, are fairly large. Moreover, the estimated probabilities correspond to the orderings between profiles, in that better performances are more likely for those profiles with the higher functioning levels associated with the respective measure. This provides support that the association of functioning levels to measures is correct.

For these measures, higher scores indicate better performance. The sample first,

second and third quartiles, which serve as upper bounds for the respective score categories, are as follows. For ADAS-Cog Delayed Recall, they are: 5, 7, 9; ADAS-Cog Word Recognition: 3, 5, 7; AVLT Trial 6: 1, 3, 4; AVLT List B: 2, 3, 4; Boston Naming: 22, 26, 28; Categorical Fluency (average of Vegetable and Animal): 10, 12.5, 15; ADAS-Cog Number Cancellation: 0, 1, 2; and Digit Symbol: 26, 35, 42.

For Trails A and B, normal mixture models are instead used to estimate corresponding response distributions. The outcomes of these measures are timed, and have a wide range of scores, so that continuous distributions models are suitable. Also, due to the heterogeneity in the sample, the respective distributions are complex and multi-modal. Hence, normal mixture models are a good fit for these measures. Estimates are graphically depicted in Figures S1 through S4. The bold curves represent the average of the sampled densities, while the colored curves are actual sampled normal mixtures from the Gibbs sampler selected 1000 iterations apart. The colored curves are included to give graphical indication of the variability in estimation.

The response distributions corresponding to each of Trails A and B are well distinguished. As performance is timed, for these measures lower scores indicate better performance. From the estimation results, lower scores are relatively more likely for those having all associated functioning levels, as would be expected. Also, note the peaks at 150 and 300 seconds respectively for response distributions associated with not having all the associated functioning. These are the worst possible scores for Trails A and B, respectively, and these peaks confirm that it is much more likely to observe these values for subjects that do not have all of the corresponding functioning levels. In sum, the estimation results indicate that the response behavior of subjects is consistent with the

cognitive specifications in the model.

Classification results, and implications for model validation: Classification results also indicate how well a model fits. Ideally, classification for each subject results in posterior probability mass for state membership concentrating on one state; in other words, the probability after observing responses from a subject for state membership is near 1 for one state, and 0 for all others. This indicates that response behavior is consistent across measures towards one state, as would be expected if the model were correctly specified. In this analysis, for the most part, posterior probability mass settled on one or two states, as reflected in Figures S5 and S6, indicating good model fit to the response data. Reasons why classification results were not always decisive include possible issues with reliability of the NP tests, or the limitations of model fit in terms of how the specified functions adequately describe performance. Still, we think that a main reason is the lack of replication of measurement in the battery. While all the states in the model can be statistically distinguished through at least one measure from the analyzed battery, administering only one measure is not always sufficient to decisively classify between two states, particularly when mid-range scores are observed. Not all measures from the full ADNI battery were used, partly to avoid the modeling of joint, multivariate distributions of test scores derived from the same administration of a measure, and also to concentrate the analysis on the modeling of the functions identified in Table 1.

Confounding of profiles, and computation of probabilities of functioning in the presence of such confounding: For the present model, performance levels for the functions can for the most part be statistically distinguished from the NP test battery under consideration, no matter the response pattern. The one exception is cognitive

flexibility, where for certain response patterns, it cannot always be ascertained what its performance level is for a subject. Cognitive flexibility is an executive function, and so is generally tested in conjunction with other functions, such as seen in Table 1. Classifying performance levels for such functions can be more problematic, as there can be confounding due to impairments with other functions.

As an example, note that given its associated cognitive profile, a subject in State 14 is only expected to perform well on ADAS-Cog word recognition and AVLT List B. For Categorical Fluency and Trails B, the two measures that tap into cognitive flexibility, expected performance is poor. This follows due to the associated lower level functioning with word fluency and perceptual motor speed, regardless of how well a subject functions with respect to cognitive flexibility. Hence, from the model, it cannot be determined if a subject has high or low functioning with cognitive flexibility.

Because of such confounding, determining the probability of a subject having a high performance level for cognitive flexibility was conducted as follows. Note that each collection of statistically indistinguishable profiles is represented by a single state in the model. For each state, the value of the proportion of these profiles that are associated with the high performance level is determined. If the association of a profile to state is one to one, note this proportion value is 1. This proportion value is multiplied by the posterior probability value that a subject belongs to the corresponding state. After doing this for each state, the resultant products are summed, to obtain an overall probability of the subject being at that level. For instance, suppose that a subject is in State14 and in State 11 with probability 0.5 each. Given that two profiles are confounded for State 14, with only one of them indicating higher performance level with cognitive flexibility, the

proportion of such profiles is 0.5. Moreover, note that State 11 is not associated with the high performance level. Hence, the probability that would be assigned for cognitive flexibility is 0.5*0.5 + 0*0.5= 0.25. The assumption here is that profiles that are confounded are equally likely to be true, which is in line with the adoption of a uniform prior probability of state membership. It is possible to assess the inferential impact of this assumption by comparing results derived from differential weighting of profiles that are confounded, to gauge sensitivity.

REFERENCES

1. Tatsuoka C: **Data analytic methods for latent partially ordered classification models.** *Journal of the Royal Statistical Society, Series C (Applied Statistics)* 2002, **51**:337-350. See also **Corrigendum** 2005, **54**:465-467.

2. Jaeger J, Tatsuoka C, Berns S, Varadi F, Czobor P, Uzelac S: **Associating functional recovery with neurocognitive profiles identified using partially ordered classification models.** *Schizophr Res* 2006, **85**:40-48.

3. Jaeger J, Tatsuoka C, Berns SM, Varadi F: **Distinguishing neurocognitive functions in schizophrenia using partially ordered classification models.** *Schizophr Bull* 2006, **32**:679-691.

4. Ishwaran H, James LF: **Approximate Dirichlet process computing in finite normal mixtures: Smoothing and prior information.** *J Comp Graph* 2002, **97**:1154-1166.

5. Tatsuoka C, Varadi F, Jaeger J: **Latent partially ordered classification models and normal mixtures.** *Journal of Educational and Behavioral Statistics*, in press.

6. Geweke J: **Evaluating the accuracy of sampling-based approaches to the calculation of posterior moments.** In *Bayesian Statistics 4.* Edited by Bernardo JM, Berger J, Dawid AP, Smith AFM. Oxford, UK: Oxford University Press; 1992

Table S1. Multinomial response probability estimates

Means of multinomial probability estimates									
		With high functioning for all associated functions				Without high functioning for all associated functions			
Measure	Score Direction	1st quartile	2nd quartile	3rd quartile	4th quartile	1st quartile	2nd quartile	3rd quartile	4th quartile
ADAS DeRecall	lower = better	0.784	0.187	0.018	0.010	0.099	0.299	0.385	0.217
ADAS WordRec	lower = better	0.437	0.346	0.154	0.064	0.024	0.153	0.296	0.527
AVTOT6	higher = better	0.023	0.074	0.148	0.756	0.458	0.373	0.106	0.063
AVTOTB	higher = better	0.142	0.233	0.283	0.342	0.363	0.313	0.251	0.073
BNTTOTAL	higher = better	0.027	0.204	0.349	0.420	0.527	0.388	0.066	0.019
CATAVG	higher = better	0.011	0.099	0.386	0.504	0.426	0.354	0.163	0.057
ADAS NumCan	lower = better	0.346	0.356	0.225	0.073	0.029	0.053	0.463	0.454
DIGITSCOR	higher = better	0.023	0.218	0.348	0.411	0.557	0.337	0.096	0.010

Standard deviations of multinomial probability estimates									
		With high functioning for all associated functions				Without high functioning for all associated functions			
Measure	Score Direction	1st quartile	2nd quartile	3rd quartile	4th quartile	1st quartile	2nd quartile	3rd quartile	4th quartile
ADAS DeRecall	lower = better	0.045	0.042	0.016	0.009	0.019	0.024	0.025	0.020
ADAS WordRec	lower = better	0.029	0.035	0.030	0.026	0.023	0.059	0.056	0.059
AVTOT6	higher = better	0.018	0.037	0.035	0.049	0.026	0.026	0.017	0.016
AVTOTB	higher = better	0.031	0.035	0.037	0.036	0.033	0.034	0.032	0.023
BNTTOTAL	higher = better	0.021	0.039	0.037	0.033	0.041	0.047	0.032	0.016
CATAVG	higher = better	0.010	0.039	0.049	0.048	0.029	0.032	0.028	0.022
ADAS NumCan	lower = better	0.021	0.022	0.020	0.014	0.028	0.047	0.104	0.102
DIGITSCOR	higher = better	0.012	0.029	0.031	0.031	0.038	0.036	0.029	0.009

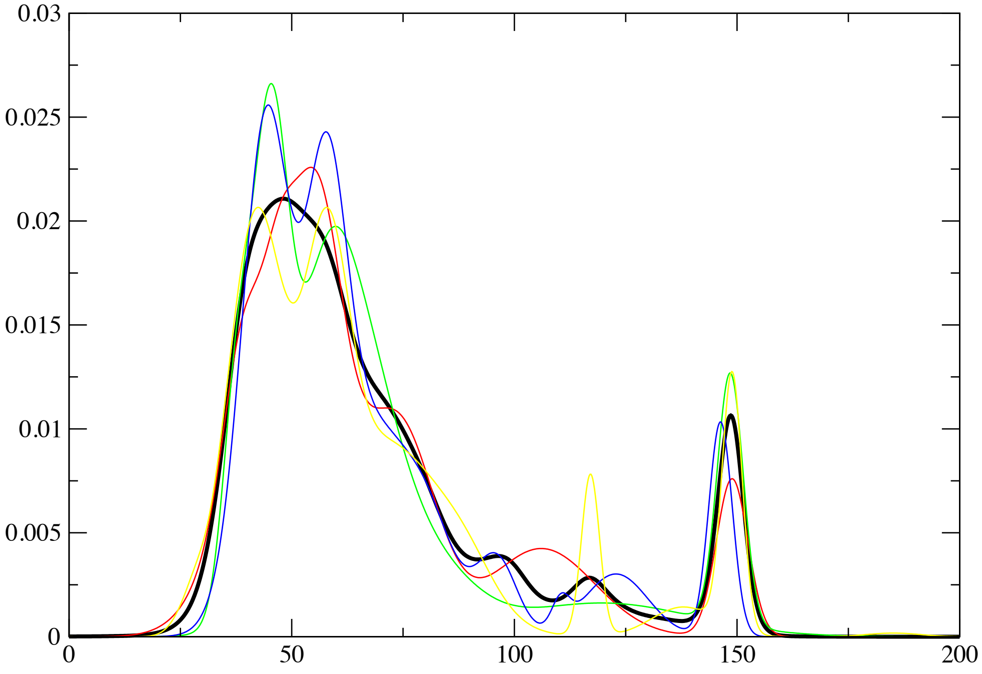

Figure S1. Estimated normal mixture response distribution for Trails A, without high functioning for all associated functions. Lower scores indicate higher proficiency. Bold curve is average density across simulations, colored curves are from a select sample. y-axis: density function value, x-axis: score value in seconds.

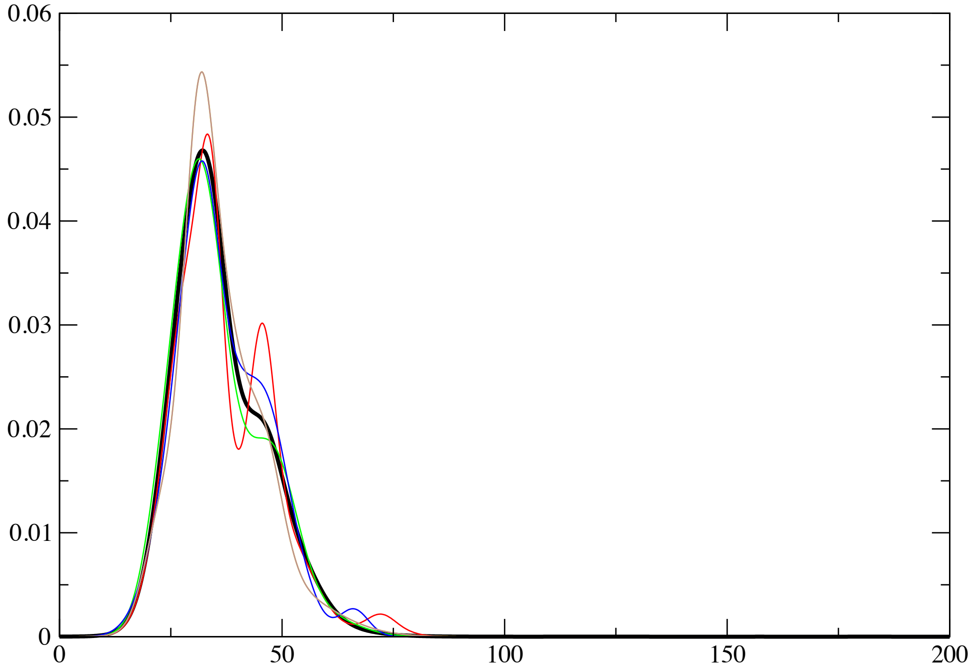

Figure S2. Estimated normal mixture response distribution for Trails A, with high functioning for all associated functions. y-axis: density function value, x-axis: score value in seconds.

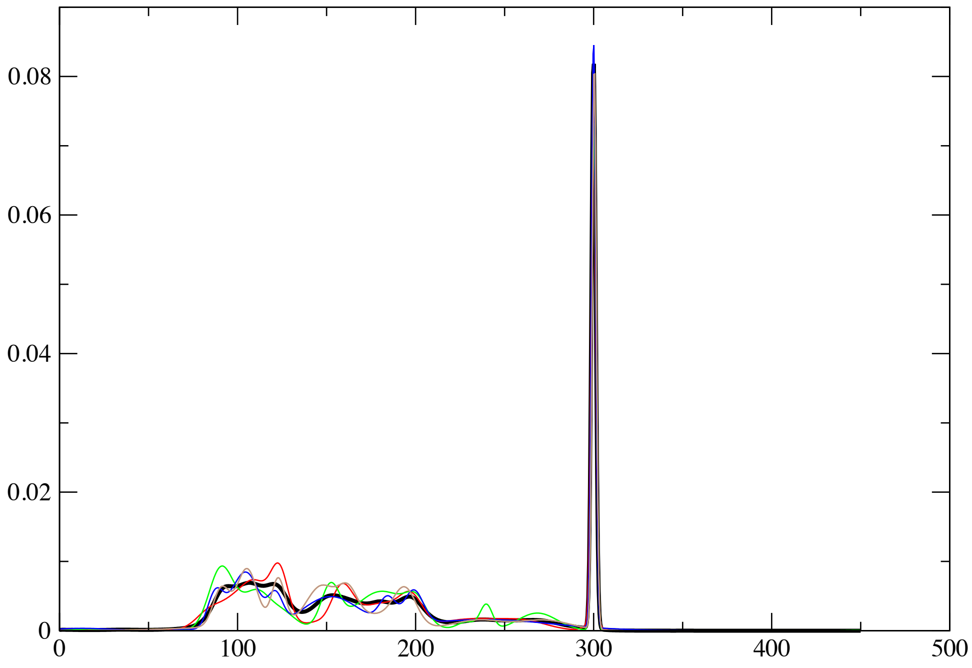

Figure S3. Estimated normal mixture response distribution for Trails B, without high functioning for all associated functions. Lower scores indicate higher proficiency. y-axis: density function value, x-axis: score value in seconds.

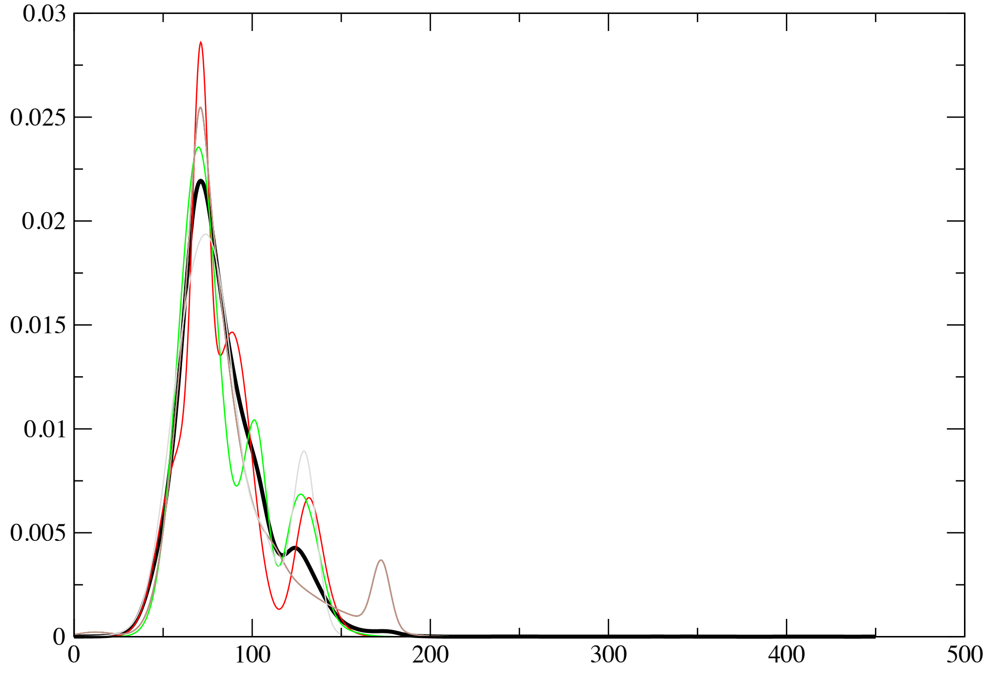

Figure S4. Estimated normal mixture response distribution for Trails B, without high functioning for all associated functions. y-axis: density function value, x-axis: score value in seconds.

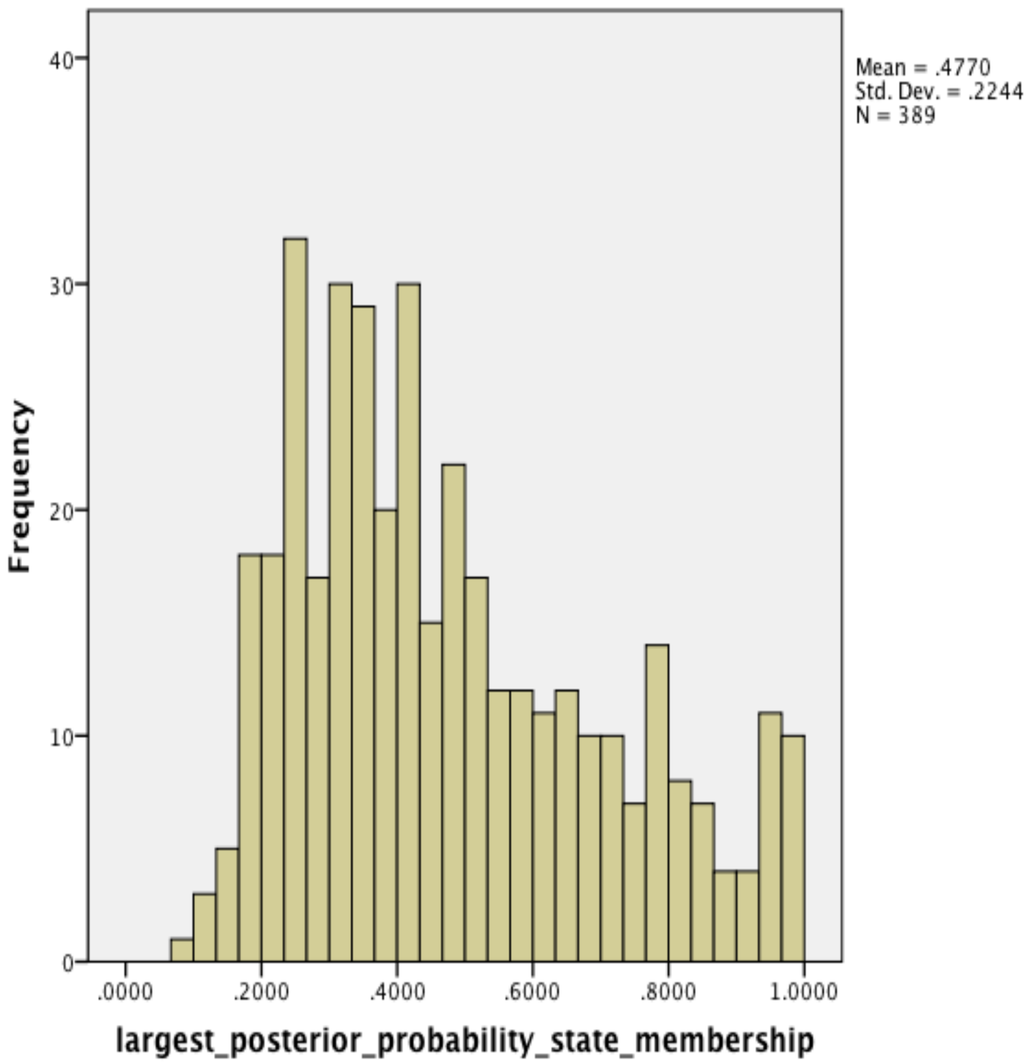

Figure S5. Largest posterior probability values of state membership at baseline

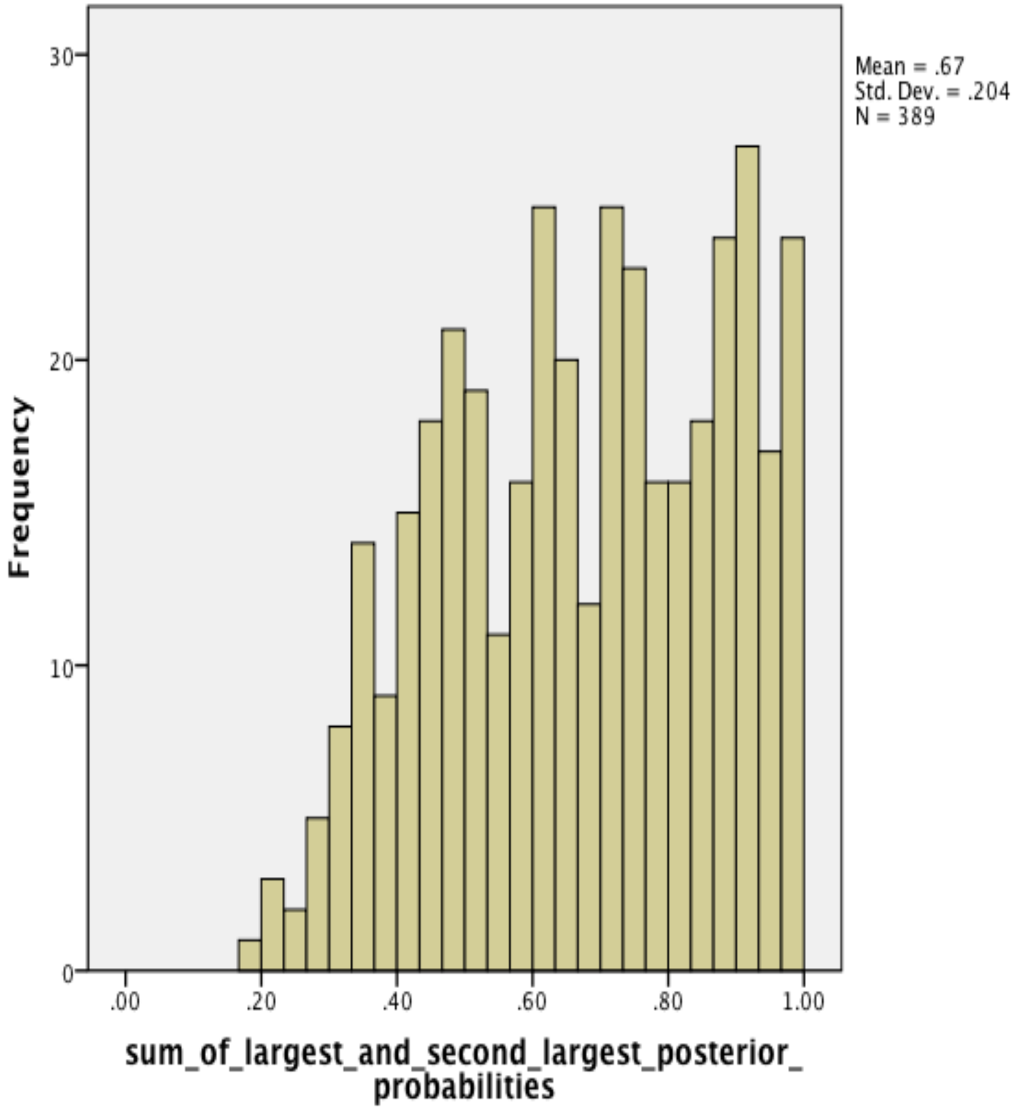

Figure S6. Sum of the largest and second largest posterior probabilities values of state membership at baseline
